# Home-Based Resistance Training for Older Subjects during the COVID-19 Outbreak in Italy: Preliminary Results of a Six-Months RCT

**DOI:** 10.3390/ijerph17249533

**Published:** 2020-12-19

**Authors:** Jacopo Antonino Vitale, Matteo Bonato, Stefano Borghi, Carmelo Messina, Domenico Albano, Sabrina Corbetta, Luca Maria Sconfienza, Giuseppe Banfi

**Affiliations:** 1 IRCCS Istituto Ortopedico Galeazzi, Via Riccardo Galeazzi 4, 20161 Milan, Italy; matteo.bonato@grupposandonato.it (M.B.); carmelomessina.md@gmail.com (C.M.); albanodomenico@icloud.com (D.A.); sabrina.corbetta@unimi.it (S.C.); io@lucasconfienza.it (L.M.S.); banfi.giuseppe@hsr.it (G.B.); 2 Department of Biomedical Sciences for Health, Università degli Studi di Milano, Via Pascal 36, 20122 Milano, Italy; stefano.brg94@gmail.com; 3 Department of Biomedical, Surgical and Dental Sciences, Università degli Studi di Milano, Via Luigi Mangiagalli 31, 20133 Milan, Italy; 4 Sezione di Scienze Radiologiche, Dipartimento di Biomedicina, Neuroscienze e Diagnostica Avanzata, University of Palermo, Via del Vespro 127, 90127 Palermo, Italy; 5 Vita-Salute San Raffaele University, Via Olgettina 58, 20132 Milan, Italy

**Keywords:** aging, sarcopenia, risk of fall, exercise, DXA, magnetic resonance imaging

## Abstract

Background. The aim of this study was to evaluate the effect of a six-month home-based resistance-training program on muscle health and physical performance in healthy older subjects during the unique condition of home confinement caused by the COVID-19 pandemic. Methods. This was a randomized-controlled study that enrolled older participants that were allocated to either an experimental group performing the six-months exercise prescription (EXE) or a control group (CON). At the beginning (PRE), and after 6 months (POST), participants were assessed for muscle strength, balance, gait assessment and body composition by dual energy X-ray absorptiometry and magnetic resonance imaging. Normality distribution of data was checked with the D’Agostino and Pearson test and changes between PRE and POST were assessed by paired Student’s *t*-test while percentage and absolute changes between groups at POST were tested by unpaired *t*-test. Results. Nine participants were included for the final analysis: EXE, *n* = 5 (age: 66 ± 4; BMI: 27.5 ± 3.7) and CON, *n* = 4 (age: 71 ± 9; BMI: 24.2 ± 4.1). Significant PRE-to-POST changes were observed in the EXE group only in the chair-stand test (+19.8%, *p* = 0.048 and ES:1.0, moderate) and in total fat mass (+5.0%, *p* = 0.035 and ES:1.4, large) with no between-group differences. Moreover, EXE had significantly higher absolute thigh CSA values than CON at POST (14.138 ± 2977 vs. 9039 ± 1015, *p* = 0.0178, ES = 1.7). No other within- and between-group differences were detected. Conclusions. The home-based resistance-training program during the lockdown period, caused by the COVID-19 outbreak, determined only within-group improvement in lower limb muscle strength but not in muscle mass and composition in older subjects. Home confinement may partially explain the increase in total body fat due to a reduced daily PA regime and altered diet pattern.

## 1. Introduction

The increase in humans’ longevity has resulted in an increased incidence of late-life chronic diseases and, consequently, the identification of non-invasive strategies to treat and prevent these negative chronic conditions, such as sarcopenia, has been a challenge in recent years. Nowadays, the primary interventions to counteract muscle decline in older people are non-pharmacological treatments and, in this context, physical activity (PA) and exercise represent the most powerful and effective methods [1,2,3]. To confirm this, in the last 20 years, several leading international organizations have recognized the ability of PA to ameliorate the growing burden of chronic disease. The American College of Sports Medicine (ACSM) proposed the concept of “Exercise is Medicine^®^”, highlighting the fundamental importance of PA for health [4] and recently, in 2020, the World Health Organization (WHO) published the new WHO 2020 guidelines on PA and sedentary behavior [5]. The authors recommended regular muscle-strengthening activity for all age groups and, in addition, older adults should do, as part of their weekly PA, varied multicomponent exercise at moderate or greater intensity on three or more days a week in order to enhance functional capacity and prevent falls [5]. In particular, it is well known that resistance training is one of the most effective strategies to prevent the deterioration of muscle strength, mass and function [6,7] by stimulating the increase in muscle protein synthesis and muscle fiber hypertrophy, leading ultimately to an increase in skeletal muscle mass and strength, and preventing disability, frailty and risk of falls in elders [8,9]. Nevertheless, one of the crucial challenges regarding resistance exercise interventions for older subjects is to promote training in a suitable setting, also ensuring the highest possible compliance by the subjects. Time and space are limited with group exercise programs and the distance between the subject’s home, and places of exercise may represent an impediment and, inevitably, participation and compliance rates are negatively influenced [10]. To ensure higher adherence to training, new and low-cost strategies, such as home-based training, have been proposed [11]. Such programs diminish the need for transportation and facilities and a recent meta-analysis by Kis et al. [12] demonstrated that home-based training is a low-cost, safe and effective exercise option to increase muscle strength and function in healthy and non-healthy older adults.

Towards the end of December, a new coronavirus (SARS-CoV-2) emerged in the city of Wuhan, the capital of the Chinese province of Hubei, causing a respiratory infectious disease with fatal consequences in the worst cases [13,14]. The outbreak has spread widely and rapidly throughout the world [15] and, on 11 March 2020, the WHO declared the outbreak of a new coronavirus disease, named COVID-19, a pandemic [16]. Between 9 March 2020 and 3 May 2020, the Italian Government adopted strict containment measures to avoid the virus spread and a possible collapse of the Italian health care system [17]. The Italian population was placed in home confinement for almost 2 months with permission only to leave home for limited and documented purposes (e.g., health reasons or buying food) and several activities were temporarily prevented, including universities and schools, restaurants, gyms and sport centers [18,19]. As a negative consequence of the home confinement, an increased time spent at home and a greater sedentary behavior were observed [20]. This scenario, unfortunately, may lead to a prolonged disuse of the skeletal muscles, which is related to the loss of functional ability in older subjects [21].

Although the effect of different kinds of home-based PA interventions in older subjects have been reported [12,22], to the best of our knowledge, the execution of a training program in a condition of home confinement during the COVID-19 outbreak has not yet been proposed. Therefore, the general aim of this randomized controlled trial (RCT) was to evaluate the effect of a six-months home-based resistance-training program on muscle health and physical performance in healthy older subjects during the lockdown period determined by the COVID-19 outbreak in Italy [23]. Specifically, as primary objective, we analyzed the PRE-to-POST improvement in the chair-stand test (CST) after six-months of home-based resistance training program. We hypothesized that the home-based PA program would determine positive effects on muscle strength and muscle function but not on body composition (i.e., fat- and fat-free mass). The rationale was that home-based exercise could prove to be a new safe and effective intervention method for preventing or improving muscle loss, thus enhancing quality of life among the older population during the pandemic.

## 2. Materials and Methods

### 2.1. Study Design

This was a six-month RCT that was conducted at the IRCCS Istituto Ortopedico Galeazzi (IOG; Milan, Italy), in accordance with the CONSORT statement [24] for RCT studies, between November 2019 and September 2020. The study was approved by the Ethical Committee of Vita-Salute San Raffaele University (ref. n.: 124/INT/2019) and all procedures were performed in compliance with laws and regulations governing the use of human subjects (Declaration of Helsinki). The study protocol was registered at clinicaltrials.gov (ClinicalTrials.gov Identifier: NCT04172285). All subjects received explanation of purpose, methods, potential risks and benefits of the study and written informed consent was obtained from all participants. As a primary objective, we compared the improvement in CST after six-months of home-based resistance training program. As secondary objectives, we compared changes from baseline to follow-up and between EXE and CON on physical performance and muscle and body composition.

### 2.2. Participants’ Screening

Patients were recruited from the outpatient Clinic of the Endocrinology and Diabetology Service of IOG, upon invitation by the caring physician, through advertisements with posters in the hospital and online through hospital social media channels. Potential participants were screened for eligibility using the following inclusion criteria: Caucasian ethnicity, aged between 60 and 80 years old, sedentary behavior and no physical exercise training ongoing, cognitive integrity, and ability to ambulate autonomously without walking aids. Exclusion criteria were: Body Mass Index (BMI) < 18.5 or >40.0, active smoking, history of cancer, a previously implanted pacemaker, recent fracture or orthopedic surgery within the past 6 months, neurological or orthopedic pathological conditions potentially affecting movement, diagnosis of balance disorder, diabetes, neoplasia, kidney or liver failure. Participants who met the above inclusion criteria were randomized with a 1:1 ratio to either an experimental group that performed an exercise intervention (EXE), or to a control group (CON) that was instructed not to perform any kind of physical activity and to follow their habitual daily routines. A computer-generated randomization list of random numbers was used for patient allocation. Allocation was concealed since both patients and investigators did not know which arm of the study the subjects would be assigned to.

### 2.3. Exercise Prescription

Subjects of EXE were instructed and familiarized on the execution of the resistance training protocol by three formed investigators (J.A.V, M.B, S.B.), experts in exercise prescription in older population, before the beginning of the experimental procedures. The first session of the resistance-training program was performed at home with the presence and supervision of the investigators to verify the subjects’ independence to exercise execution. Therefore, EXE performed four home-based resistance-training sessions per week for 24 consecutive weeks (total training sessions: 96). Each session consisted of 5 min of warm-up, 45 min of resistance exercise intervention and 5 min of cool-down. In detail, the warm-up included light dynamic movements and exercises for all the body areas and joints (e.g., walking and skipping on site), including upper/lower limbs (e.g., shoulder and elbow circles or half squat) back (e.g., twists and side bending), and neck (e.g., 3-axis movements). The central part of the training included resistance exercises both for lower and upper body muscles: all exercises were performed utilizing only the participants’ body weight or a few extra weights when needed (i.e., small (≃500 g) or large (≃1500 g) water bottles). A monopodalic balance exercise was also included in the training session. The kind of exercise, the number of sets and repetitions, the seconds of resting period and the exercise progression are detailed in Table 1. The cool-down training phase consisted of light static stretch exercises, lasting between 25 and 40 s, for the main body muscles, previously involved during the training sessions (neck, back and upper and lower limbs).

The training adherence was monitored, as suggested [25], both by weekly phone calls and by the compilation of a daily diary. All subjects wrote in the diary the training date, the duration of the entire session, the number of sets and repetitions for each exercise. The minimum acceptable adherence to the training program was 75% to include subjects for final analysis.

### 2.4. Clinical Assessments

Before the beginning of the experimental procedures (PRE) and at the end of the six months of home-based exercise intervention (POST), all subjects completed the following clinical evaluations: 1. Anthropometric assessment; 2. Whole-body dual energy X-ray absorptiometry to assess total body fat-free and fat mass; 3. Thigh magnetic resonance imaging to determine the cross-sectional muscle area; 4. Risk of fall assessment through the mini-Balance Evaluation Systems Test; 5. Strength assessment by means of: a. the chair stand test; b. handgrip strength test; c. dynamometers to assess maximal isometric strength of knee flexors and extensors muscles.

#### 2.4.1. Anthropometric Assessment

Height was measured to the nearest 1 cm and body mass to the nearest 0.5 kg (Seca 217, Vogel and Halke, Hamburg Germany). Body-mass index (BMI) was calculated using the standard formula (weight in kg divided by height in meters squared).

#### 2.4.2. Whole-Body Dual Energy X-ray Absorptiometry

DXA is the reference standard technique in clinical practice for the assessment of body composition (bone, fat and muscle mass) because of low costs and wide availability [3,26,27,28]. Total fat mass and lean mass at arms, limbs, trunk, and as total body was measured by dual-energy X-ray absorptiometry (DXA) (Hologic QDR-Discovery W densitometer; Hologic Inc., Bedford, MA, USA). Subjects’ Appendicular Skeletal Muscle Mass Index (ASMMI), indicating the amount of muscle in the upper and lower limbs, corrected by the individuals’ square of the height, was considered as a valid value of muscle mass. Specifically, the most widely accepted cut-off values for ASMMI, obtained with the whole-body DXA-scan, are 5.5 Kg/m^2^ in women and 7.0 Kg/m^2^ in men, respectively [29].

#### 2.4.3. Magnetic Resonance Imaging Scan

Dixon magnetic resonance imaging (MRI) sequences on the axial plane at the middle third of the thigh was performed with a 1.5T MR system (Avanto, Siemens Medical Solution, Erlangen, Germany) to precisely quantify the thigh cross sectional area (CSA) [30,31]. Cross sectional T1-weighted and DIXON sequences were obtained at the middle thigh, with 15 slices of a 5-mm thickness that were acquired covering a total length of 7.5 cm. The segmentation of the thigh muscles was performed, with the use of the ImageJ free software [32] by an expert investigator (C.M.), for each slice in which the muscle-tendon junction of the gluteus maximus muscle was clearly visible. The whole muscle area was selected as a single unit while femur, subcutaneous fat and blood vessels were excluded from the segmentation. The CSA of thigh muscles is expressed in cm^2^.

#### 2.4.4. Strength Assessment

Participants warmed up prior to performing the strength tests by cycling for 5 min on a cycle-ergometer and were familiarized with the testing procedures; all strength tests were supervised by two expert investigators (M.B. and S.B.).

#### 2.4.5. Chair Stand Test

The 30 s chair stand test (CST) [33] was performed to evaluate lower limb strength. The test has been shown to provide valid and reliable data on leg strength in older adults [33]. The CST is administered using a chair with a seat height of 17 inches (43.2 cm) and the subject has to start the test in a sitting position with crossed arms at the wrists and head on the chest. At the “go” signal by an expert investigator (S.B.), the participant had to rise in a full standing position and then return to the initial sitting position. The subject has to complete as many full stands as possible in 30 s and higher scores indicate higher lower limb strength values.

#### 2.4.6. Hand Grip Strength Test (HGS)

HGS is a measure of the maximum isometric force that a hand can squeeze and it is widely used because very easy and inexpensive [34]. The handgrip strength was measured three times for each hand alternatively, starting with the dominant hand, using a dynamometer (Hand Grip Meter 6103, Tanita, Tokyo, Japan) to the nearest 0.1 kg. Participants performed the test in a sitting position with their elbows fixed at a 90-degree angle with their wrist in a comfortable position. The subjects were asked to squeeze the dynamometer as hard as possible for three seconds. Participants rested 60 s between each trial. Verbal encouragement was provided to ensure maximal effort. The average of three trials for each hand was calculated and used for statistical analysis.

#### 2.4.7. Maximal Isometric Strength (MIS) of Knee Flexors and Extensors Muscles

Knee flexors and extensors MIS was measured, for both lower limbs alternatively, using a belt-stabilized dynamometer (Sauter FK 1k, Sauter GmbH, Balingen, Germany) with subjects in a setting position and the knee placed at 90° flexion. Measurements using a belt-stabilized dynamometer have been shown to be valid and reliable in older subjects when compared to the standard method of isometric knee muscle strength assessments using isokinetic dynamometers [35,36,37]. MIS was specifically recorded in Newtons (N) and three repetitions were performed for each limb, first for knee extensors and then for knee flexors. The participants were instructed to remain seated in an upright position and the upper limbs were placed on the bed to support the body and prevent a fall. A resting period of 30 s was given after each repetition. Verbal encouragement was provided to ensure maximal effort. The average of three trials for each limb was calculated and used for statistical analysis.

#### 2.4.8. Balance and Gait Assessment

The Mini-BESTest, a newer and shorter version of the original BESTest [38], is a valid tool for the assessment of dynamic balance and gait deficits in older people [39]. The Mini-BESTest showed very good inter-rater and test–retest reliability when assessed in a sample of people with increased risk of falling [40]. This instrument contains 14 items evaluating four different aspects of dynamic balance: anticipatory postural adjustments, postural responses, sensory orientation, and balance during gait. Each item has a score from zero (lower score) to two (higher score) and the total maximum score is 28, with higher scores indicating better balance. The Mini-BESTest took approximately 12–15 min to administer and it was performed in the same gait analysis laboratory, at the same length of time, with subjects wearing the same comfortable sport shoes.

### 2.5. Statistical Analysis

Quantitative variables were expressed as mean ± standard deviation (SD) and 95% Confidence Intervals (CI). The normality of the distribution of the outcome measures for the two groups was checked using graphical methods and the D’Agostino and Pearson test. Because the tested variables were normally distributed, parametric tests were used for analysis. Participants’ characteristics at baseline of EXE and CON were compared by unpaired *t*-test and the Fisher exact test was used to compare the proportion of males and females between EXE and CON groups. To pursue the primary aim of the study, changes between PRE and POST were assessed by paired Student’s *t*-test while absolute and percent changes between groups at POST were tested by unpaired *t*-test. The magnitude of change after home-based exercise was analyzed by means of a modified statistical spreadsheet [41]. The spreadsheet calculates the standardized differences or effect size (ES). Threshold values for ES statistics were: ≤0.2, trivial; >0.2, small; >0.6, moderate; >1.2, large; ≥2.0, very large [42]. The level of significance was set at 0.05. Statistical analysis was performed using Graph Pad Prism Software, version 8.0 for Windows (Graph Pad Software, San Diego, CA, USA).

## 3. Results

### 3.1. Study Population

**Figure 1:**
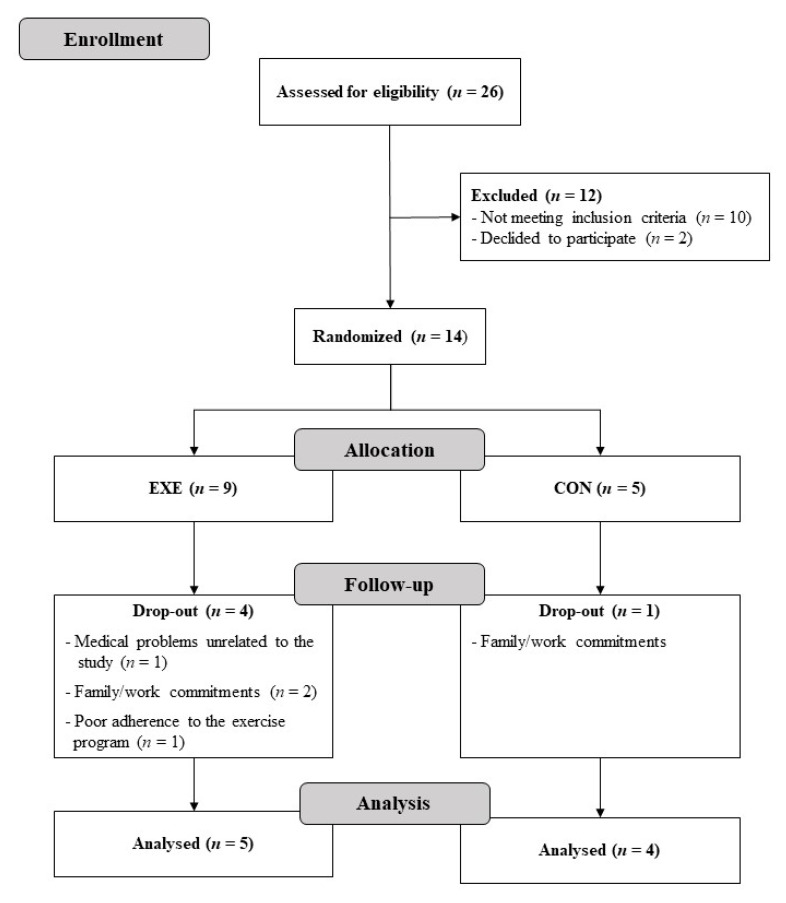
Participants flow diagram.

Twenty-six participants were screened and 14 were eligible: nine were allocated in the EXE group and five in the CON group. Twelve participants were non-eligible for the study because they did not meet the inclusion criteria (*n* = 10) or declined to participate (*n* = 2). Four participants of the EXE dropped out during the training intervention due to medical problems (*n* = 1), family commitments (*n* = 2) or non-adherence to the training program (*n* = 1). One participant of the CON group dropped due to work commitments. Nine participants were, therefore, included for the final analysis (EXE, *n* = 5; CON, *n* = 4). No adverse events were observed during the study. Figure 1 shows the flowchart of the subject’s screening and participation.

Table 2 shows the Mean ± SD and 95% CI baseline characteristics of the nine participants who completed the study. There was no difference between the two training groups in age, height, body mass, BMI, handgrip strength and Mini-BESTest. Significant difference was observed in ASMMI, caused primarily by the randomization process, resulting in an unbalanced male distribution across the two training groups. All participants had a sedentary lifestyle, defined as physical activity for less than 2 days/week for less than 20 min per session.

### 3.2. Physical Performance Evaluation

Table 3 shows the mean ± SD and 95% CI of the physical function test performed at PRE and POST. Significant PRE-to-POST changes were observed in the EXE group only in the chair stand test (*p* = 0.048; ES: 1.0, moderate). Neither significant POST changes from PRE, nor significant absolute and percentage change differences between groups were observed for the other parameters.

### 3.3. Body Composition Evaluation

**Figure 2:**
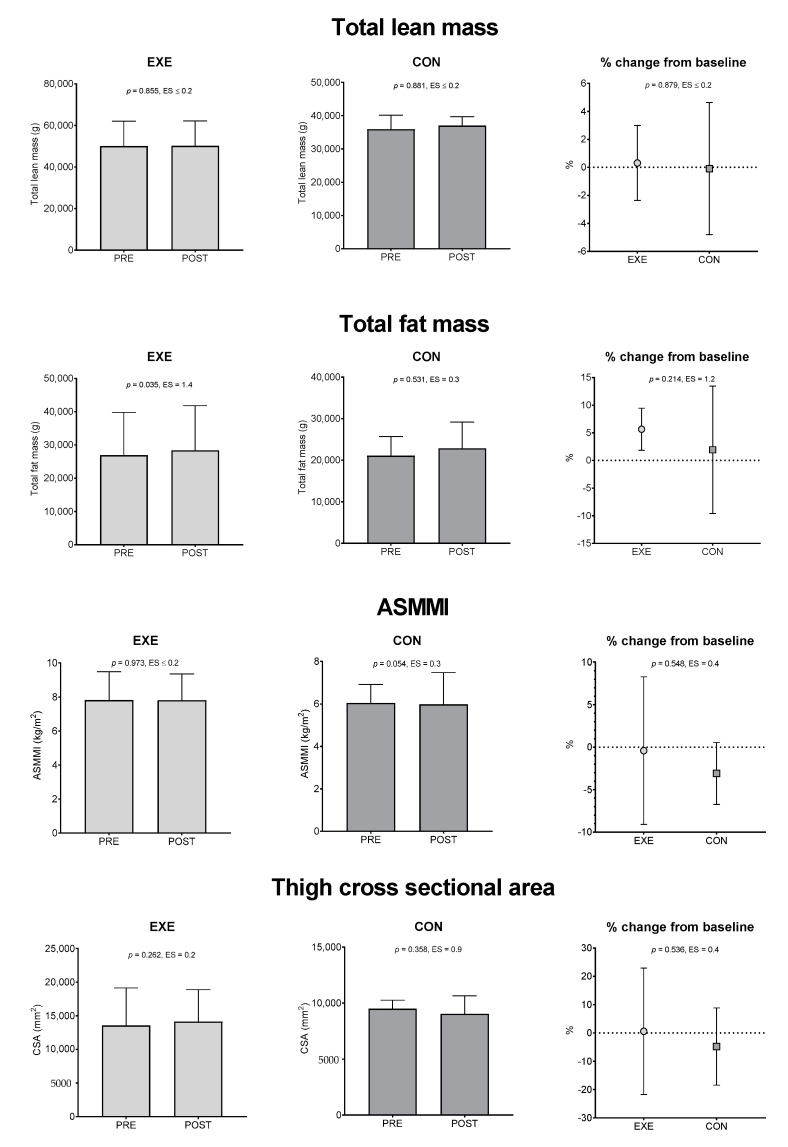
Total lean mass, total fat mass, appendicular skeletal muscle mass index and cross-sectional area results. Values are expressed as mean ± 95% CI. Data were assessed by paired *t*-test (differences between PRE and POST within groups) and unpaired *t*-test (differences between groups). Level of significance was set at 0.05. The magnitude of changes was analyzed by Effect Size. EXE: experimental group; CON: control group; ASMMI: appendicular skeletal muscle mass index; *p*: *p*-value; ES: effect size.

Figure 2 shows Mean ± SD and 95% CI of the body composition parameters at PRE and POST. Significant PRE-to-POST changes were observed in the EXE group only in total fat mass (*p* = 0.035; ES: 1.4, large) and, in addition, we observed that EXE had significantly higher thigh CSA values than CON at POST (14,138 ± 2977 vs. 9039 ± 1015, *p* = 0.0178, ES = 1.7). Finally, neither significant POST changes from PRE, nor significant absolute and percentage change differences between groups were observed for the other parameters.

## 4. Discussion

To the best of our knowledge, this is the first study that examined the effect of a home-based resistance training program on muscle strength and composition during the lockdown period due to the COVID-19 outbreak in Italy. Our initial hypotheses were partially confirmed: we observed that the six-months training program led to a significant PRE-to-POST improvement only in CST with no between-groups differences for this variable; the other strength-related variables did not show any within- or between-group difference. In addition, EXE showed an increase in total body fat from PRE to POST (and this trend was observed in CON too, but the statistical significance was not reached) and also had significantly higher thigh CSA absolute values with respect to CON at POST.

The present results must be interpreted in the context of home confinement due to the COVID-19 outbreak [17]. Italy is indeed one of the most impacted countries by the virus and the Italian Ministry of Health shows that more than 600,000 people have been infected with approximately 42,900 deaths [43]. The WHO officially declared COVID-19 a pandemic in March 2020 [23] and, due to this critical emergency, the Italian Government adopted, between the 9 March 2020 and 3 May 2020, strict containment measures to avoid the virus spread [17]. Consequently, the Italian citizens experienced a quarantine period, and many daily activities were largely limited. The home confinement led to general health issues and had a large impact on several aspects of human health [44,45]: prolonged staying home has been associated with a sedentary lifestyle, modified diet patterns and higher levels of stress. Home confinement also made it more difficult to reach the new 2020 WHO recommendations of regular aerobic and muscle strengthening PA [5,46,47]. Therefore, in this context, home-based resistance training may represent a valid and alternative strategy to mitigate physical inactivity in the older population.

One of the strengths of this study is that the training program was an ecologic home-based intervention, with no request of any specific gym instrumentation and associated costs. In line with this, previous studies reported improvements in older subjects’ muscle strength and function capacity using home-based resistance training modality [48] and a recent meta-analysis showed that this kind of intervention is a safe and effective exercise option to specifically increase lower body muscle strength in older subjects [12]. These results are in line with our study. In our RCT, the mean adherence to the training intervention, evaluated by means of weekly phone calls and by a training diary filled by the subjects, was 84.8%, and this indicates that EXE performed the resistance training program with consistency [49]. All subjects wrote in their diary the training date, the duration of the entire session, the number of sets and repetitions for each exercise. The training program determined a significant +19.8% PRE-to-POST increase in CST for EXE (*p* = 0.048, ES:1.0) with no between-group differences for this variable while HGS, MIS of knee flexors and extensors muscles and the Mini-BESTtest did not display any significant variation. Similarly, CON did not modify strength values at POST. For what concerns body composition, only few within- and between-group differences were detected: EXE registered a significant PRE-to-POST increase in total body fat (+5%; *p* = 0.035, ES:1.4) with no between-group differences and, in addition, EXE also had significantly higher thigh CSA values than CON at POST (14,138 ± 2977 vs. 9039 ± 1015, *p* = 0.0178, ES = 1.7).

Recently, Cunningham et al. [50] observed that incidental and planned PA decreased in people with social distancing and, in addition, the adult population during the lockdown period decreased by 60% the time spent for PA and increased by 42% the sedentary time [51]. The subjects of EXE constantly performed the home-based exercise program however, two of the six months of the training intervention took place during the lockdown period and this may explain both the increase in lower limb muscle strength in the CST, due to the exercise intervention, and the increase in body fat, due to a reduced daily PA regime and altered diet pattern. CON did not significantly change the body composition; however, a trend toward an increase in total body fat was observed in this group too. At baseline, the two study groups were homogenous for BMI, age, handgrip strength and balance ability (i.e., mini BESTest) but they differed in ASMMI, with EXE having higher values than CON (*p* < 0.045 and ES: 1.4, large). This difference was to be attributed to the fact that CON included only females, while EXE had three males and two females. Nonetheless, none of the participants were classified as sarcopenic on the basis of the ASMMI (cut-off value for female: 5.5 Kg/m^2^ and male: 7.0 Kg/m^2^) [29].

Our study suffers from limitations. First, the relatively small sample size did not allow for drawing firm conclusions but, nevertheless, we were able to detect the efficacy of the home-based exercise program on lower limb muscle strength in the EXE group. Second, men were underrepresented in our population. Although assignment to either the EXE or CON group was randomized, men were all allocated to the EXE group, resulting in unbalanced distribution between the two groups. Third, dietary intake was not restricted or controlled, which might have influenced the study outcomes, especially total body fat. Fourth, we did not collect specific data on the sedentary and PA behavior of both groups (excluding training for EXE) during the home confinement period, and these data would have helped to better interpret the results on muscle strength and body composition.

## 5. Conclusions

It is essential for older people to practice resistance training at home, especially during home confinement, to limit harmful muscular and functional decline. The present study highlights that participants allocated to the EXE group improved lower limb strength during the lockdown period, and these benefits in muscle strength were likely associated with the home-based resistance training program. However, noteworthy is that no between-group differences in strength values were observed. Therefore, the present study provides only limited evidence on the effect of home-based training on lower limb strength. The home confinement determined by the COVID-19 outbreak in Italy, which led to higher sedentary levels, together with the lack of a specific diet regimen, also caused an increase in total body fat. With this scenario, home-based resistance training should be promoted as alternative, valid, and cost-effective modality of exercise to favor physical health during home confinement. Our study forms the basis for the design of larger interventional studies in order to assess the feasibility and the efficacy of long-term, possibly self-managed, exercise approaches in older subjects.

## Figures and Tables

**Table 1:** Description of the resistance exercises included in the central part of the home-based training session.

Exercise Type		Number of Sets	Number of Repetitions	Resting Time	Exercise Progression	Load
Sitting on- and standing from a chair	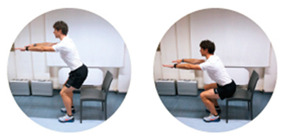	4	15	60–90 s	increase of sets (up to *n* = 5) and/or repetitions (up to *n* = 20) and/or squat exercise (no chair) and/or use of two bottles as extra-weights	Body weight
Leg adduction from a standing position	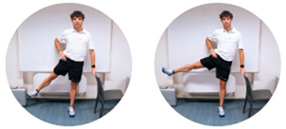	4	15	60–90 s	increase of sets (up to *n* = 5) and/or repetitions (up to *n* = 20) and/or use of sport anklets as extra-weights	Body weight Sport anklets
Leg abduction from a standing position	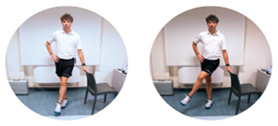	4	15	60–90 s	increase of sets (up to *n* = 5) and/or repetitions (up to *n* = 20) and/or use of sport anklets as extra-weights	Body weight Sport anklets
Calf exercise from a sitting position	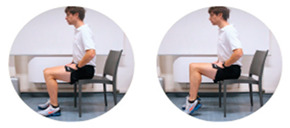	4	15	60–90 s	increase of sets (up to *n* = 5) and/or repetitions (up to *n* = 20) and/or execution from a standing position	Body weight
Monopodalic balance exercise from a standing position with external support	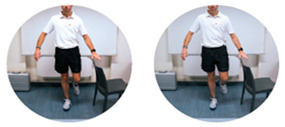	3	30 s	60–90 s	increase of sets (up to *n* = 4) and/or time of work (up to 45 secs) and/or execution with closed eyes	Body weight
Side raises from a sitting position	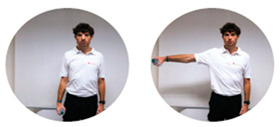	3	12	60–90 s	increase of sets (up to *n*= 4) and/or repetitions (up to *n* = 20) and/or execution from a standing position and/or use of two larger bottles as extra-weights	Bottles
Biceps curl from a sitting position	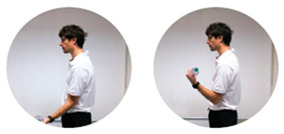	3	12	60–90 s	increase of sets (up to *n* = 4) and/or repetitions (up to *n* = 20) and/or execution from a standing position and/or use of two larger bottles as extra-weights	Bottles
Triceps curl from a sitting position	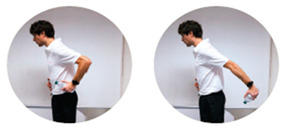	3	12	60–90 s	increase of sets (up to *n* = 4) and/or repetitions (up to *n* = 20) and/or execution from a standing position and/or use of two larger bottles as extra-weights	Bottles

The following general recommendations were considered for the exercise progression: 1. Week 1–6: begin with the suggested values for sets and repetitions (highlighted in the table); 2. Week 7–12: increase only the number of sets but not the number or repetitions; Week 13–18: maintain the increased number of sets and increase the number of repetitions; Week 19–24: maintain the increased number of both sets and repetitions and modify the exercise (when possible), such as: from sitting to squatting (exercise 1) or from open eyes to closed eyes (exercise 5) or using a larger bottle of water as extra weight (exercise 6). The exercise progression was always supervised by an expert investigator.

**Table 2:** Baseline characteristics of the 9 participants who completed the study. Values are expressed as number of participants (%) or as mean ± SD and (95% CI). Data were compared between groups by unpaired *t*-test and Fisher exact test. EXE: experimental group; CON: control group; BMI: body mass index; ASMMI: appendicular skeletal muscle mass index; *p*: *p*-value; ES: effect size; n.a.: not applicable.

Variable	Total (*n* = 9)	EXE (*n* = 5)	CON (*n* = 4)	*p*	ES
Male (*n*, %)	3, 33%	3, 60%	0, 0%	0.167	n.a.
Age (years)	68 ± 7 (62.9–73.1)	66 ± 4 (60.8–71.2)	71 ± 9 (56.5–84.5)	0.342	1.25
Height (m)	1.64 ± 0.08 (1.55–1.71)	1.67 ± 0.09 (1.56–1.78)	1.61 ± 0.09 (1.42–1.74)	0.196	0.6
Body mass (kg)	70 ± 16 (57.4–81.7)	77 ± 17 (55.9–98.2)	60 ± 6 (50.4–69.2)	0.095	1.0
BMI (kg/m^2^)	26.0 ± 4.0 (22.9–29.1)	27.5 ± 3.7 (22.9–32.1)	24.2 ± 4.1 (17.6–30.7)	0.237	0.9
Handgrip strength (kg)	27.4 ± 6.8 (22.2–32.8)	30.4 ± 7.5 (21.17–39.7)	23.8 ± 4.3 (16.9–30.66)	0.236	0.9
ASMMI (kg/m^2^)	7.0 ± 1.4 (5.9–8.1)	7.8 ± 1.3 (6.1–9.5)	6.0 ± 0.6 (5.2–6.9)	0.045	1.4
Mini-BESTest	25 ± 3 (23–27)	25 ± 3 (22–28)	25 ± 4 (19–30)	0.981	<0.2

**Table 3:** Chair stand test, handgrip strength, Mini-BESTest, thigh extensors and flexors strength results. Values are expressed as mean ± SD and (95% CI). Data were assessed by paired *t*-test (differences between PRE and POST within groups) and unpaired *t*-test (differences between groups). Level of significance was set at 0.05. The magnitude of changes was analyzed by Effect Size. EXE: experimental group; CON: control group; **Δ: change PRE to POST**; *p*: *p*-value; ES: effect size.

Variable	Within-Group Differences								Between-Group Differences			
	EXE				CON				EXE	CON		
	PRE	POST	*p*	ES	PRE	POST	*p*	ES	Δ ± SD	Δ ± SD	*p*	ES
Chair-stand-test (rep)	14 ± 2 (11–16)	16 ± 3 (13–19)	0.048	1.0	14 ± 4 (7–19)	15 ± 3 (10–20)	0.215	0.3	2.6 ± 2.1 (0.1–5.2)	1.5 ± 1.9 (−1.5–4.5)	0.441	0.5
Handgrip strength (kg)	30.4 ± 7.5 (21.2–39.7)	28.2 ± 9.7 (16.1–40.2)	0.417	0.3	23.8 ± 4.3 (16.9–39.7)	23.3 ± 3.6 (17.6–29.0)	0.468	≤0.2	−2.3 ± 5.6 (−9.2–4.6)	−0.5 ± 1.3 (−2.4–1.5)	0.556	0.4
Mini-BESTest (score)	25 ± 3 (22–28)	26 ± 1 (25–27)	0.260	0.3	25 ± 4 (18.6–30.4)	25 ± 3 (20.2–29.3)	0.637	≤0.2	1.2 ± 2.1 (−1.4–3.7)	0.3 ± 0.9 (−1.3–1.8)	0.425	0.4
Thigh extensors strength (*n*)	333 ± 96 (214–453)	329 ± 93 (213–445)	0.825	≤0.2	242 ± 109 (68.5–415.3)	264 ± 102 (102.2–426.4)	0.591	0.2	−4.4 ± 42.3 (−57.0–48.2)	−1.5 ± 48.2 (−78.2–75.2)	0.926	≤0.2
Thigh flexors strength (*n*)	176 ± 49 (115–237)	178 ± 51 (115–241)	0.931	≤0.2	164 ± 43 (96.1–231.3)	140 ± 44 (70.3–208.7)	0.09	0.6	2.1 ± 49.9 (−59.9–64.1)	−35.3 ± 14.9 (−59.1–11.6)	0.272	0.7
